# Dr. Kinglake, on Delivering the Placenta

**Published:** 1800-08

**Authors:** Robt. Kinglake

**Affiliations:** Chilton super Poldon


### *To the Editors of the Medical and Physical Journal.*

GENTLEMEN,

IN a late Number of your Journal* appeared a paper from Dr. Squire, containing strong and pointed animadversions on a doctrine detailed in a former part of your publication† by Mr. Peck, recommending the expediency of an early extraction of the placenta. The opinions in question, on this subject, differ extremely; and on no occasion, perhaps, could the almost universally applicable advice of the father of Phaethon, "*Medio tutissimus ibis,*" be more advantageously regarded than in the present instance.

While an implicit compliance with the admonition of uniformly proceeding to extract the placenta by manual operation, if retained longer than fifteen minutes after the expulsion of the child, though neither hæmorrhage nor any other urgent circumstance should indicate its necessity, might, in general, be deemed an undue anticipation of the seasonable efforts of uterine power, and, in some cases, actually prove perniciously precipitate; a rigid conformity to the precept inculcating indefinite delay, if no hæmorrhage occur, would lead to a practice invariably pusillanimous, and, occasionally, more precarious and disastrous, than can be fairly charged to the account of the other extreme.

The worst effect likely to result from a premature but well conducted extraction of the placenta is, aggravation of pain during the operation; the probability of subsequent evil can scarcely be an object of dread, as the stimulus of *vacuity* on the extrusion of the placenta, is generally sufficient to excite the

---

* Vide No. XV. p. 459. † Vide No. XIII. p. 221.

the uterus to an approximation of its parietes, which, at once, effectually obviates hæmorrhage, and appeases inordinate irritation. The mischief that may accrue from indeterminate delay, consists in the depressing anxiety which it is liable to induce in the patient; the debilitating continuance of inadequate uterine irritation; incidental hæmorrhage from partial detachment of the placenta, and the alarm inevitably occasioned by being eventually compelled to perform under the distress of urgent necessity, what might have been more seasonably executed with the composure and address inspired by prudent precaution.

My opinion on this controversial point, has arisen solely from long and extensive experience, and fully warrants me in adopting an intermediate course of practice.

Directing my conduct not by any abstract rule, but exclusively by the circumstances of the case; after the birth of the child, and as soon as the divided extremities of the funis have been secured by ligature, my operative attention is incessantly engaged in the removal of the placenta, neither by violence, nor in negligence of uterine effort; but, by keeping the funis moderately stretched, varying the line of draught from the perpendicular to an anterior, posterior, and lateral direction, cautiously co-operating with every contraction of the uterus, by a guarded increase of extension. In this manner, invariable success has attended my endeavour within half an hour; no untoward circumstance has ever occurred; the funis has in no instance been ruptured, nor that event even endangered, when it possessed its natural firmness; no hæmorrhage has been induced; no complaint of augumented pain, nor any sort of subsequent inconvenience. This practice is now so perfectly familiar to me, that I have the utmost confidence in its adoption; and feel equally justified in its pursuit, by principles of *science* and *humanity*. *Scientifically*, because it appears to afford Nature the peculiar aid which moderate tension only is adapted to render, in obviating the enfeebling flaccidity which occurs in a comparatively vacant state of the uterus after the exclusion of the child, and in at once gently exciting and reinforcing the natural disposition to uterine contraction. *Humanely*, because the exertion proposed can inflict no injury, and by insuring a definite expulsion, all anxiety is terminated, and contingent disaster precluded.

From the preceding statement, it will be inferred, that my practice has more analogy to that proposed and vindicated by the advocates for early extraction, than to that recommended and espoused by the partisans for unlimited delay; but, that while it differs from the former in superceding the necessity for direct operative detachment of the placenta, (that ultimate occasion having never presented in my own practice, in the course of many hundred cases). it varies from the latter, in proceeding immediately to assist the uterus by moderate co-operation, in exonerating itself of what may be considered as a morbid state of excitement.

It has twice occurred to me to be called to assist gentlemen, who, adopting the doctrine of patiently awaiting the spontaneous exertions of the uterus for the expulsion of the placenta, had, in both instances, allowed it to remain upwards of twelve hours, during the whole of which time, inefficient pain had recurred at intervals. The mode of assistance herein proposed, was immediately employed; and, in both cases, within half an hour, the placenta was safely removed, without superinducing any sensible degree of additional irritation; nor did hæmorrhage or any other inconvenience supervene.

It is uncertain how long the placenta, in the instances alluded to, might have been retained; probably, until the inadequate contractions of the uterus had effected a partial detachment, and the consequent hæmorrhage had compelled the *patient* practitioners to furnish manual aid. The mental suffering of the patients would have been cruelly aggravated by longer delay, and no compensation could have accrued from superior advantages by ultimate natural expulsion.

Dr. Squire has adduced a formidable legion of authorities in support of his opinion,* and appears, very consistently, to have assumed no small portion of confidence from the respectable mass of suffrage he has cited. It would be equally to calumniate Nature and the defenders of her competency, to declare, that artificial aid is *uniformly* necessary in placental delivery. In fact, it is but very rarely, that art need be resorted to, either to avert or remedy error. Indeed, the mere possibility of accidental deviations from natural rectitude, affords the only justification that can be assumed for the ordinary practice of midwifery. It not unfrequently occurs, that women, even in civilized nations, safely undergo parturition in solitude; nor has the obstetric art been familiarly known in countries where vicious refinements have not perverted the order of Nature, and introduced the various physical and moral evils of licentious civilization.

As the practice of midwifery is not uniformly necessary, and only occasionally required to countervail constitutional degeneracy and mal-conformation, chiefly originating from the corrup-

* Vide Medical and Physical Journal, No. XV. pp. 482—3.

rupt inftitutions and habits of art; it is peculiarly important, ftrictly to regard the legitimate objects of affiftance, that fuitable aid may be correctly and promptly rendered when indifpenfably requifite; and not be ufeleffly, or pernicioufly obtruded, on what is exclufively the work of natural power.

The ordinary progrefs of parturition, under natural prefentation, cannot be advantageoufly affifted by art; due patience will ultimately evince the fidelity of Nature to her engagement.

The fupport of the perineum is a precaution more worthy of the kind office of a kindred female, than of the folemn parade of obftetric fkill. The benefit afforded in fecuring againft its laceration, by moderating and regulating the uterine impulfe, is imaginary. Scarcely a calculable probability exifts of that event occurring; and the refiftance ufually oppofed to it, could not prevent the accident, while it may injure the uterus by counteracting its protrufive efforts. The expulfion of the placenta fafely admits of definite acceleration; and, in natural parturition, its feafonable removal may be truly faid to conftitute the fole utility of midwifery practice.

In a large majority of cafes the placenta would, no doubt, be fpontaneoufly extruded; but it will not unfrequently happen that much anxious protraction of time, alarming hæmorrhage, and unavailing irritation, may be happily prevented by its early removal.

The mode propofed, approaches to an imitation of Nature in fuftaining that tenfive ftate of the uterus which preceded the expulfion of the child, and which would be continued in fome degree after birth by the infantile ftruggles, were the funis to remain unfevered.

A firm perfuafion, founded on ample *experience*, of the perfect fafety and expediency of this practice, is my only motive for fubmitting it to public confideration. It need be but tried to be approved, and its uniform adoption would end all ambiguity and poffible hazard relating to *placental deilvery.* I am,

Gentlemen,

Your's, refpectfully,

ROBT. KINGLAKE.

*Chilton super Poldon,*

*July* 12, 1800.

